# Mitochondrial haplogroup U is associated with a reduced risk to develop exfoliation glaucoma in the German population

**DOI:** 10.1186/1471-2156-11-8

**Published:** 2010-01-28

**Authors:** Christiane Wolf, Eugen Gramer, Bertram Müller-Myhsok, Francesca Pasutto, Bernd Wissinger, Nicole Weisschuh

**Affiliations:** 1 Centre for Ophthalmology, Institute for Ophthalmic Research, Molecular Genetics Laboratory, Tuebingen, Germany; 2 University Eye Hospital, Julius-Maximilians-University, Wuerzburg, Germany; 3 Max Planck Institute of Psychiatry, Munich, Germany; 4 Institute of Human Genetics, Friedrich-Alexander-University Erlangen-Nuremberg, Erlangen, Germany

## Abstract

**Background:**

Various lines of evidence demonstrate the involvement of mitochondrial dysfunction in the pathogenesis of glaucoma. Therefore, mitochondrial DNA is a promising candidate for genetic susceptibility studies on glaucoma. To test the hypothesis that mitochondrial haplogroups influence the risk to develop glaucoma, we genotyped 12 single-nucleotide polymorphisms that define the European mitochondrial DNA haplogroups in healthy controls and two German patient cohorts with either exfoliation glaucoma or the normal tension subgroup of primary open angle glaucoma.

**Results:**

Mitochondrial haplogroup U was significantly under-represented in patients with exfoliation glaucoma (8.3% compared with 18.9% in controls; p = 0.004).

**Conclusions:**

People with haplogroup U have a lower risk to develop exfoliation glaucoma. Our results substantiate the suggestion that mitochondrial alterations have an impact on the etiology of glaucoma.

## Background

Glaucoma is among the leading causes of blindness in elderly people of western European ancestry [1]. In the last ten years a few genes and a number of unsolved gene loci for open angle glaucoma have been identified based on the analyses of mendelian forms of glaucoma. These however only comprise a small fraction of the total glaucoma patient population. None of these genes or loci has been shown to have a relevant impact on the glaucoma population as a whole. The strong hereditary component in glaucoma most likely results from multigenic inheritance involving multiple susceptibility genes [2].

Diverse pathophysiological mechanisms are discussed to be involved in glaucoma including vascular dysregulation, excitotoxicity, autoimmunity and oxidative stress [3-6]. The latter is relevant to neuronal damage in the glaucomatous retina and optic nerve head since it triggers mitochondrial dysfunction and leads to retinal ganglion cell (RGC) death in a cell culture model [7]. The pathogenic role of reactive oxygen species in glaucoma is supported by various experimental findings, such as an increase of the oxidative stress markers superoxide dismutase, catalase, and glutathione peroxidase in the aqueous humor of patients with primary open angle glaucoma and exfoliation glaucoma [8,9]. A significant correlation between oxidative DNA damage in the trabecular meshwork and intraocular pressure (IOP) increase and visual field defects was observed in glaucomatous patients [10]. In an independent study, a significant reduction of mitochondrial respiratory function in peripheral blood cells was observed in glaucoma patients when compared to age-matched healthy controls [11]. Another study showed that the exposure of RGCs to elevated hydrostatic pressure resembling an elevated intraocular pressure results in altered mitochondrial fission [12].

The involvement of mitochondrial dysfunction in glaucoma can also be certified by maternal inheritance. Several studies have shown that a maternal family history is more frequent than a paternal history in glaucoma [13-16] which suggests a contribution of mitochondrial DNA (mtDNA) to glaucoma pathogenesis. In fact, an increased frequency of nonsynonymous mtDNA sequence changes in patients with primary open angle glaucoma has been shown [17]. However, this report describes a relatively small number of patients from a restricted ethnic population and confirmation in other populations is still lacking.

MtDNA is exposed to a higher mutation rate compared to nuclear DNA as it is not protected by histones. Furthermore, the repair mechanisms in mitochondria are not as efficient as those in the nucleus [18,19]. Among the numerous mutations that have accumulated in mtDNA during evolution there are several ethnic specific single nucleotide polymorphisms (SNPs) that enable the definition of discrete and region specific subdivisions of the human population called mitochondrial haplogroups (mtHgs). More than 90% of European mtDNAs belong to nine haplogroups (H, V, U, K, J, T, W, I and X), which are highly specific for Western Eurasia [20]. Specific mtHgs might reflect functional differences in energy metabolism and were therefore linked with human diseases like neurodegenerative disorders [21] or cancer [22]. Polymorphisms in mtDNA might also represent modifier factors as has been shown in Leber's hereditary optic neuropathy (LHON) where mtHgs contribute to the phenotypic expression of mtDNA mutations [23-25].

The relationship of mtHg distribution and glaucoma has not been subject of comprehensive studies so far. One study was based on the phylogenetic network for European mtDNA [26]. The authors genotyped 140 patients with primary open angle glaucoma and 75 healthy controls but could not detect a significant difference of mtHgs frequencies between patients and controls [27]. A second study in the Saudi-Arab population [28] compared the prevalence of region specific mtHgs in 552 healthy controls with a patient cohort that was composed of three different glaucoma subtypes (49 patients with primary open angle glaucoma, 29 cases with primary angle closure glaucoma and 29 cases with exfoliation glaucoma). Like in the first study, the authors were not able to identify significant differences between patients and controls for all mitochondrial haplogroups tested except for the 29 primary angle closure glaucoma patients. The authors however admitted that the statistical significance reached for this patient group might have been accidental due to the small number of cases.

The aim of our study was to evaluate the distribution of mtHgs in two German cohorts with exfoliation glaucoma (n = 157) and the normal tension subgroup of primary open angle glaucoma (n = 272). The frequencies of ten common European mtHgs in both cohorts were compared with those in a control group (n = 249) of German origin.

## Results and Discussion

We studied the mitochondrial haplogroup distribution among 272 patients with the normal tension subgroup of primary open angle glaucoma (NTG) and 157 patients with exfoliation glaucoma (XFG). Two hundred and forty-nine individuals matched for age, gender and ethnicity and with exclusion of glaucoma served as control group.

Ten major European haplogroups (HV, H, I, J, K, T, U, V, W and X) were observed in both patient groups and in controls (Table 1). All frequencies observed in the control group were consistent with previous studies of European populations [29-31].

**Table 1:** Haplogroup distribution in patients and controls

	Controls (n = 249)	NTG (n = 272)					XFG (n = 157)				
**mtHg**	**f**	**f**	**p**	**OR**	**L95**	**U95**	**f**	**p**	**OR**	**L95**	**U95**

**H**	0.450	0.434	0.725	0.937	0.663	1.324	0.491	0.475	1.177	0.790	1.756
**U**	0.189	0.132	0.093	0.656	0.410	1.049	0.083	**0.004**	0.388	0.204	0.737
**T**	0.108	0.147	0.193	1.418	0.845	2.379	0.115	0.872	1.065	0.570	1.991
**J**	0.068	0.063	0.860	0.910	0.459	1.804	0.070	1.000	1.028	0.476	2.222
**K**	0.056	0.099	0.075	1.850	0.956	3.578	0.083	0.312	1.515	0.704	3.265

**O**	0.040	0.0					0.006				
**V**	0.024	0.033					0.051				
**W**	0.020	0.015					0.032				
**HV**	0.016	0.018					0.038				
**I**	0.016	0.018					0.013				
**X**	0.012	0.018					0.019				

O, unclassified mitochondrial haplogroups; f, frequency; OR, odds ratio; L95, confidence interval 95% lower bound; U95, confidence interval 95% upper bound. Odds ratios were calculated only for haplogroups with a frequency >5%. The only significant p-value is in bold.

When comparing the frequencies of mitochondrial haplogroups between cases and controls, we observed that haplogroup U was clearly under-represented in XFG patients (8.3% compared to 18.9% in controls). Statistical analysis with Fisher's exact test revealed that this difference was significant (p = 0.004).

In NTG patients, haplogroup U was slightly under-represented (13.2 % compared to 18.9% in controls) whereas haplogroup T was slightly over-represented (14.7% compared to 10.8% in controls). However, these differences were not significant (Table 1). Either mitochondrial haplogroups are not associated with this specific disease entity or NTG is genetically more complex than XFG and therefore larger sample sizes would be required to detect subtle differences in the distribution of mtHgs. As has been shown by Samuels and co-workers [32] subtle changes in haplogroup frequency will require cohorts that should be at least twice as large as our patient cohorts. This holds true especially for low-frequency mtHgs which we excluded a priori from statistical analysis.

There is growing evidence that certain mtHgs are associated with distinct diseases. In Europe, some mtHgs have been found to be protective against neurodegenerative diseases. Haplogroups J and K are underrepresented in Parkinson disease [33] and haplogroup U is underrepresented in female patients with Alzheimer disease [34]. However, the contribution of a specific mitochondrial haplogroup to disease mechanisms is yet difficult to explain. Whereas it seems unlikely that single SNPs alter the efficiency of mitochondrial energy production enough to cause a specific disease, there is evidence that certain mtDNA genetic backgrounds can modify primary mutations: Several independent studies support a role for haplogroup J in the expression of certain LHON mutations [23-25].

We cannot rule out that other than the genotyped mtDNA variants are present in our patients as we did not evaluate the whole mitochondrial genome. Further studies should focus on this point as it may provide more insight into the association of mtHgs with glaucoma.

So far, only two studies dealt with association of mtHgs and glaucoma, and both are not ideally suited to be compared with our results. The first study analyzed patients of British origin [27] and the second focussed on patients from Saudi Arabia [28]. Due to the different geographical distribution of mtHgs our results from a middle European population cannot be directly compared with the study performed in Saudi Arabs. The British study, on the other hand, focussed on primary open angle glaucoma patients and not exfoliation glaucoma or normal tension glaucoma.

Apart from the mtHg distribution, our study provides a second line of evidence that substantiates a possible involvement of mitochondrial DNA in glaucoma pathogenesis. Within the NTG patients, a maternal history of glaucoma (17%) is more prevalent than a paternal history (8%) (data not shown). This difference is statistically significant (p = 0.01). Within the XFG patients, 18% have an affected mother whereas only 10% report an affected father (data not shown). This difference does not reach significance level (p = 0.13) but indicates a higher prevalence for maternal history in XFG glaucoma. Although several sources of bias might influence these results (recall bias; closer offspring contact with mothers than fathers; greater life expectancy of females that increases the rate of affected women in a late onset disease such as glaucoma), a higher prevalence of maternal history in glaucoma has been reported repeatedly [13-16] and alternative mechanisms for this observation (e.g. imprinting) have not yet been identified.

Although the association of haplogroup U with XFG reached significance level, it is important to point out that the power of our study is still relatively limited. In Germany, almost half of any study population belongs to the predominant haplogroup H, thus limiting the power to detect an association among the remaining haplogroups due to a rigorous reduction of these sample sizes.

## Conclusions

Our data indicate that mitochondrial haplogroup U is associated with a reduced risk to develop exfoliation glaucoma in the German population. Further studies in larger cohorts are needed to confirm this observation. Sequencing the entire mitochondrial genome might lead to the identification of other than mtHg-defining variants that contribute to the disease phenotype. The incorporation of data from genome-wide association studies might reveal synergistic interactions of mtHgs with other genes.

## Methods

### Study samples

This study adhered to the tenets of the Declaration of Helsinki. Written informed consent was obtained from all subjects and the study was approved by the ethics committees of the Universities of Tuebingen, Wuerzburg and Erlangen-Nuremberg.

The entire study population comprised 678 subjects of German origin including 272 patients with normal tension glaucoma (NTG), 157 patients with exfoliation glaucoma (XFG) and 249 healthy controls.

The NTG cohort comprised 177 women and 95 men. The mean age of individuals was 65.0 ± 13.0 years (median 67 years). NTG was defined by (1) the presence of typical glaucomatous optic neuropathy with corresponding visual field loss, (2) open drainage angles on gonioscopy, (3) absence of a secondary cause for glaucomatous optic neuropathy, such as previously elevated IOP after trauma, a period of steroid administration or uveitis, and (4) IOP measures of untreated NTG continuously 21 mmHg or lower on repeated diurnal testing (five readings between 8:00 AM and 6:00 PM). The median of readings was required to be 21 mm Hg or less, with no reading above 24 mm Hg and no more than one reading of 23 or 24 mm Hg. IOP readings were correlated with corneal thickness. Patients did not have evidence of high myopia or congenital ocular abnormality and had no other cause than glaucoma for disc changes and visual field loss. Disc size and parameters were evaluated by confocal examination (Heidelberg Retina Tomograph). To minimize interobserver variability, more than 95% of patients were examined by the same ophthalmologist. All patients had a long term follow-up to ensure diagnosis of NTG with a maximum of certainty. In large part, NTG patients underwent a neurological examination to exclude an intracerebral expansion. Sonography was used to rule out aortic stenosis.

The XFG cohort comprised 89 women and 68 men. The mean age in this group was 72.5 ± 8.7 years (median 72 years). All patients diagnosed with exfoliation syndrome in this study showed manifest exfoliation deposits in the anterior chamber. All of them met the criterion of XFG as they showed intraocular pressure (IOP) exceeding 21 mm Hg and typical glaucomatous optic neuropathy with compatible visual field loss.

Ethnicity of XFG and NTG patients was assessed for the last three generations by standardized questionnaire and self-reporting. Family history information was obtained from 200 NTG patients and 157 XFG patients. This information originated from questionnaires completed by the patients themselves.

The control cohort comprised 149 women and 100 men and was matched with the patient cohorts for age, gender distribution and geographical origin. The average age in controls was 66.8 ± 13.3 years (median 68 years). Slit lamp microscopy in mydriasis was performed to exclude presence of exfoliation deposits on intraocular tissues. IOPs were in the normal range (<21 mm Hg) and they had no glaucomatous disc damage. Controls had no family history of glaucoma.

### Isolation of DNA and genotyping

DNA was extracted from peripheral blood lymphocytes using the Magnetic Separation Module I from Chemagen using DNA chemistry (Chemagic DNA Blood Kit Special; Chemagen AG, Baesweiler, Germany).

The TaKaRa LA PCR Amplification Kit (Takara Bio Inc., Shiga, Japan) was used according to the manufacturer's protocol to amplify a 12612 bp fragment of mtDNA comprising all polymorphic sites that were used to characterize and distinguish the European haplogroups H, V, HV, U, K, J, T, W, I and X (primers are given in Table 2). Specific extension primers were designed to genotype mtHg specific SNPs (Table 2). Two separate multiplex primer extension reactions (MPE R1 and MPE R2) each covering 6 informative SNPs were performed using the ABI PRISM SNaPshot Multiplex Kit (Applied Biosystems (ABI), Weiterstadt, Germany). Capillary-based electrophoretic separation of multiplex primer extension products was performed on a sequence detection system (ABI 7500). Sequence data were analyzed using ABI PRISM GeneMapper Software version 3.7. SNPs for haplogroup typing were selected as previously reported and are listed in Table 3[35,36]. Haplogroups that could not be assigned to one of the major European haplogroups with this scheme were designated as "others" (O).

**Table 2:** List of primers used for amplification of mtDNA and primer extension reactions

Reaction	Primer sequence	Nucleotide position
**LD PCR**	forward: ACAACCCTTCGCTGACGCCATA	-
	reverse: GGTGGTACCCAAATCTGCTTCC	-

**MPE R1**	AGCCATCGCTGTAGTATA	14470
	ACCTGAGTAGGCCTAGAAATAAACAT	4580
	(A)_8_CTACACGACACGTACTACGTTGTAGC	7028
	(A)_12_GTTTATTACTCTTTTTTGAATGTTGTCAAA	10034
	(A)_23_CGACTCATTAAATTATGATAATCATAT	10463
	(A)_27_TACTCAAATGGGCCTGTCCTTGTAGTATAAA	15904

**MPE R2**	TGACCCCAATACGCAAAA	14766
	(A)_5_TCCATTGGTCTTAGGCCCCAA	12308
	(A)_12_TGTTAGCGGTTAGGCGTACGGC	8994
	(A)_17_GTGACTACAAAAAGGATTAGACTGA	10398
	(A)_16_CTTCCTACCACTCACCCTAGCATTACTTATATGA	4216
	(A)_28_GGCCACCTACTCATGCACCTAATTGGAAGC	9055

LD, long distance; MPE R, multiplex primer extension reaction. Nucleotide position according to the revised Cambridge reference sequence (GenBank: J01415.2).

**Table 3:** Selected SNPs for discrimination of ten common European haplogroups

Nucleotide position	H	V	HV	W	U	K	I	X	T	J
**14470**	T or A	T	T	T	T	T	T	C	T	T
**4580**	G	G or A	G	G	G	G	G	G	G	G
**7028**	C	T	T	T	T	T	T	T	T	T
**10034**	T	T	T	T	T	T	C	T	T	T
**10463**	T	T	T	T	T	T	T	T	C	T
**15904**	C	T	C	C	C	C	C	C	C	C
**14766**	C	C	C	T	T	T	T	T	T	T
**12308**	A	A	A	A	G	G	A	A	A	A
**8994**	G	G	G	A	G	G	G	G	G	G
**10398**	A	A	A	A	A	G or A	G	A	A	G
**4216**	T or C	T	T	T	T	T	T	T	C	C
**9055**	G	G	G	G	G	A	G	G	G	G

Nucleotide position according to the revised Cambridge reference sequence (GenBank: J01415.2).

### Statistical analysis

Frequencies of mtHgs in patients and controls were tested for independence using Fisher's exact test. Only mtHgs with a frequency >0.05 were tested. A p-value < 0.05 was considered significant. Multiple testing errors were corrected by the Bonferroni method. As five mtHgs were tested and the haplogroup distribution was compared with the controls among each of the two glaucoma subgroups, the significance level was reduced to α = 0.005.

Significance for deviation from a fifty-fifty-distribution of maternal and paternal disease history was calculated using a Χ^2 ^test.

## Authors' contributions

CW carried out the genotyping, performed the statistical analysis, drafted the manuscript and participated in the design of the study. EG performed the clinical examination of all patients and participated in the coordination of the study. BM-M participated in the statistical analysis. FP participated in the recruitment of control subjects. BW participated in study design and helped drafting the manuscript. NW conceived of the study, and participated in its design and coordination and helped to draft the manuscript. All authors read and approved the final manuscript.
